# Outcomes of polio eradication activities in Uttar Pradesh, India: the Social Mobilization Network (SM Net) and Core Group Polio Project (CGPP)

**DOI:** 10.1186/1471-2334-11-117

**Published:** 2011-05-10

**Authors:** William M Weiss, MH Rahman, Roma Solomon, Vibha Singh, Dora Ward

**Affiliations:** 1 Department of International Health, Johns Hopkins Bloomberg School of Public Health, 615 North Wolfe Street, Suite E8132, Baltimore, MD 21205, USA; 2 CORE Group Polio Project - India, 45/201 Heritage City, MG Road, Gurgaon, India; 3 CORE Group Polio Project - India, 106A/1, Gautam Nagar, New Delhi, India; 4 CORE Group Polio Project, 151 Ellis Street, NE, Atlanta, GA 30303, USA

## Abstract

**Background:**

The primary strategy to interrupt transmission of wild poliovirus in India is to improve supplemental immunization activities and routine immunization coverage in priority districts with a focus on 107 high-risk blocks of western Uttar Pradesh and central Bihar. Villages or urban areas with a history of wild poliovirus transmission, or hard-to-reach or resistant populations are categorized as high-risk areas within blocks. The Social Mobilization Network (SM Net) was formed in Uttar Pradesh in 2003 to support polio eradication efforts through improved planning, implementation and monitoring of social mobilization activities in those high-risk areas. In this paper, we examine the vaccination outcomes in districts of SM Net where the CORE Group works.

**Methods:**

We carried out a secondary data analysis of routine monitoring information collected by the SM Net and the Government of India. These data include information about vaccination outcomes in SM Net areas and non-SM Net areas within the districts where the CORE Group operates. Statistical analysis was used to compare, between SM Net and non-SM Net areas, vaccination outcomes considered sensitive to social mobilization efforts of the SM Net. We employed Generalized Estimating Equations (GEE) statistical method to account for Intra-cluster Correlation (ICC), and used 'Quasi-likelihood under the independence model criterion (QIC)' as the model selection method.

**Results:**

Vaccination outcomes in SM Net areas were as high as or higher than in non-SM Net areas. There was considerable variation in vaccination outcomes between districts.

**Conclusions:**

While not conclusive, the results suggest that the social mobilization efforts of the SM Net and the CORE Group are helping to increase vaccination levels in high-risk areas of Uttar Pradesh. Vaccination outcomes in CORE Group areas were equal or higher than in non-CORE, non-SM Net areas. This occurred even though SM Net areas are those with more community resistance to polio vaccination and/or are have harder-to-reach populations than non-SM Net areas. Other likely explanations for the relatively good vaccination performance in SM Net areas are not apparent.

## Background

There were an estimated 350,000 cases of wild poliovirus in 1988 [1]. In dramatic contrast, the total number of wild polio cases in all of 2009 has dropped to 1604; the global total number of 2010 wild polio cases is 618 as of 24 August 2010 [2]. Four countries are endemic for wild polio: Afghanistan, India, Nigeria and Pakistan [3]. As of 24 August 2010, there have been 30 reported cases of wild poliovirus in India compared to 236 during the same period in 2009 [2]. Almost all wild polio cases in India are from high-risk districts in western Uttar Pradesh and central Bihar [3]. The most recent primary strategy to interrupt transmission of wild poliovirus in India is to improve supplemental immunization activities (SIAs or mass campaigns) and routine immunization coverage in 107 high-risk blocks of western Uttar Pradesh and central Bihar [4]. It is widely acknowledged that grass roots social mobilization efforts are needed to reach underserved populations during SIAs and to combat rumors against polio vaccination [5-7]. The CORE Group is a US-based organization made up of health professionals, working for a variety of non-governmental organizations, who collaborate on international health and development programs [8]. In 1999, the United States Agency for International Development (USAID) launched the CORE Group Polio Project (now known as CGPP) in six countries, including India. The CGPP harnesses and synchronizes the efforts of a coalition of US-based Private Voluntary Organizations (PVOs) and their in-country offices, as well as non-governmental organization (NGOs) partners to support the polio eradication effort by providing both social mobilization and detailed local planning for vaccination services. The CGPP focuses on targeting the most inaccessible populations, whether due to cultural or physical barriers to access. The project reaches these populations by systematic enumeration and tracking of children less than five years, and through highly targeted social mobilization strategies that rely on direct personal communication with families and with informal and formal community leaders.

In India, the CGPP works in ten districts of the state of Uttar Pradesh (UP) through a consortium of the following PVOs: Adventist Development & Relief Agency (ADRA) India, Project Concern International (PCI) and Catholic Relief Services (CRS), as well as their local NGO partners. The CGPP India has a Secretariat providing coordination and technical and managerial support to field staff. The CGPP India has an extensive network of 1,325 Community Mobilization Coordinators (CMCs) who conduct social mobilization activities for behavior change related to polio vaccination. These CMCs are a part of the Social Mobilization Network (SM Net) in India that includes CORE, UNICEF, Rotary, and the Indian Government's and WHO's National Polio Surveillance Project (NPSP). The SM Net was formed in UP in 2003 to support polio eradication efforts there by doing the following: identifying high-risk areas and working with underserved communities in planning, implementing and monitoring social mobilization and other immunization activities in those high-risk areas. The three-tier network of community mobilizers (community level, block level, and district level) does the main work of the SM Net.

### Organization Of The Social Mobilization Network (SM Net)

Formed in 2003, the Social Mobilization Network (SM Net) in Uttar Pradesh, India comprises the CORE Group Polio Project (CGPP), Unicef, the National Polio Surveillance Project, and Rotary International. CGPP and UNICEF implement synchronized social mobilization activities using community level workers called community mobilization coordinators (CMCs). The SM Net developed the behavior-change communication materials, training materials, supervision structure and pay scale with uniform guidelines; these are implemented consistently across CGPP and Unicef areas. Over time, the SM Net has standardized field staff positions and functions, expanded and refined the data collected by the CMCs and incorporated increasingly focused behavior-change communication techniques.

The Community Mobilization Coordinator (CMC) interacts with families and community members at the village level. As the backbone of the SM net, s/he is assigned responsibility for mobilizing about 500 households in either a rural or urban area, and keeps records of the immunization status of all 0-5 year children in those households. During SIA (Supplementary Immunization Activity) rounds, CMCs assist vaccinators in setting up vaccination booths, organize groups of child mobilizers (*Bulawwa tollies*) and arrange for mosque and/or temple announcements. CMCs also do the following: accompany vaccinator teams to all the houses; work to convince families with an unvaccinated child (called an 'X' household) to allow their child to be vaccinated (converting an 'X' household to 'P' (denoting a house where all eligible children are vaccinated against polio); and, accompany persons of influence (influencers) during follow-up activities.

In between the SIA Rounds, the CMC carries out activities aimed at increasing OPV coverage. S/he visits houses, talking to mothers and other caregivers, dispelling their doubts or rumors about the vaccine. S/he holds mothers meetings to discuss their children's health and explains prevention and management of common illnesses. One of the most interesting activities that s/he conducts is harnessing the potential of schoolchildren. S/he conducts 'Polio classes' at schools in her/his area. In these classes, she uses various methods to get the children interested in becoming a part of the polio campaign---from poetry and painting competitions on the Polio theme, to rallies. A few children are then selected to come together as 'Bullawa tollies' (Literal translation = Calling gangs) who mobilize mothers to bring their children to the booths during SIAs.

In India, the Block is the smallest administrative unit and is made up of 100-150 villages. Within the SM Net, the Block Mobilization Coordinator (BMC) oversees social mobilization activities during (and in between) SIA rounds through supervision and mentoring of the CMCs working in the block. The BMC reports to the District Mobilization Coordinator. H/she is responsible for the following activities: capacity building of all CMCs through training; data collection and collation by CMCs; building partnerships with the Medical officer in charge of the Block Primary Health Center and other stakeholders; ensuring that routine immunizations are conducted in hard to reach areas; conducting inter-personal communications (IPC) sessions; participating in their block task force and other related meetings; organizing health camps; and, monitoring CMC vaccination booths and house-to-house vaccination visits.

The District Mobilization Coordinator (DMC) is in charge of social mobilization activities in all the CORE blocks of the district. S/He is responsible for the following activities: compilation of all block level data that are sent to the Secretariat; training; participation in District Task Force meetings; developing the joint SM Net District Communication Plan; building strong partnerships with the district government officials and other stakeholders; and, ensuring routine immunization sessions are conducted in hard to reach areas. During SIAs, the DMC monitors the quality of vaccination activities at CMC vaccination booths and during house-to-house visits. In addition, h/she updates records about the SIA and provides feedback to the Chief Medical officer, and medical officers in charge of the health centers. The District Underserved Coordinator (DUC) is in charge of planning and implementation of activities in underserved areas in the district, such as liaison with religious leaders and religious institutes and ensuring mosque announcements. The Sub Regional Coordinator (SRC) is an employee of one of the CGPP partner organizations and oversees activities in multiple districts. S/He collaborates with local government departments, non-governmental organizations, religious institutions, and other polio partners.

CMC areas are villages where the SM Net deploys CMCs. The SM Net selects these villages for additional social mobilization efforts based on past communication and operational challenges for immunizing children. Most of the CMCs are deployed in areas designated as High Risk Areas (HRAs). Jointly with key partners (Unicef, MOH and CGPP), NPSP defines the criteria for HRAs; these criteria are reviewed periodically and modified. The most recent criteria for HRAs take into account the following information: the number of wild polio virus (P1) cases during low transmission seasons since 2003; the presence of High Risk Groups (Slum dwellers/Nomads); the number of acute flaccid paralysis cases that were compatible with polio in last two years; if 40% or more of the population is Muslim; and, the percent of households that have unvaccinated children (X houses). Once an area is identified as an HRA, the SM Net arranges for CMCs to work there. A CMC has to be 18 years or more, preferably female and from the same community. The partnership periodically revises the areas designated as an HRA.

### How vaccination is done during SIAs in SM Net CMC areas

Vaccination during SIAs in SM Net areas is conducted in the following way. On Day 1 of the SIA, almost always a Sunday, fixed booths are set up where the vaccination teams are stationed. The team consists of three persons, and the third person is hired from the same community to mobilize children from houses. In CMC areas, CMCs accompany team members and mobilize children by making *bulawwa tollies *of older children from the same neighborhood (as described above). From Day 2 through Day 5 or 6, vaccination teams, called A Teams, visit about a hundred households each day to document vaccination of all eligible children. Once the vaccination team has visited all assigned households on a particular day, they count the number of households that have not had all eligible children vaccinated. These are called "X" households. The vaccination team then revisits all the "X" households to vaccinate children. The CMC accompanies the vaccination team during visits to "X" households and uses various methods to convince families to vaccinate all eligible children. If an "X" household allows all eligible children to be vaccinated, then we say that an "X" household has been converted to "P." For example, if a family refuses to allow their child to receive vaccine, the CMC may arrange for an influential person in the community to come and speak with the family and encourage vaccination. This ends the A Team effort on a particular day for the team's assigned households. There might remain a number of "X" households, however, in the section of households that the A Team passed through that day.

On the day after the A Team has passed through a section of houses, another team, called B teams, visits the households still labeled "X" after the A Team's efforts. This is the last chance during the SIA to vaccinate eligible children in "X" households through B team activities. The CMC accompanies B team members and visits all "X" houses remaining after A team to communicate to the families, the importance of OPV, and also, if required, to arrange influencer's visit. At the end of the B team activities, there might still remain a number of "X" households.

The Indian Academy of Pediatrics (IAP) has praised the efforts of SMNet in western UP for reducing the number of "X" houses during house to house immunization, increased booth coverage, and led to a reduction in 'resistant' households [5]. The IAP additionally encouraged the SM Net to continue its social mobilization efforts in UP. This is important because social resistance to polio vaccine remains a key barrier to eradication in western UP [9,10]. As the social mobilization efforts of the SM Net in western UP continue, it would be useful to more rigorously evaluate the outcomes of these efforts. While the IAP cites some evidence (above) of improved vaccination outcomes, the question arises about the value-added of the SM Net efforts: Does having a CMC provide any advantage in achieving vaccination outcomes? In the Patna Region of the State of Bihar, there is a report that vaccination outcomes in areas with CMCs are improving faster than in areas without CMCs [11]. In this paper we examine the evidence for the value-added of the SM Net in western UP.

In this paper, we examine the performance of the SM Net efforts in the CGPP districts only. We assume that performance in Unicef areas is similar because the CMC personnel structure is used in Unicef areas is the same as in CGPP areas; however, it is possible that there are differences. The objective of this study is to learn if vaccination outcomes in SM Net areas are as good or better than in non-SM Net areas within the CGPP program. We will compare vaccination efforts in areas within a block that have CMCs with non-CMC areas in the same block.

## Methods

### Study design

This study is a secondary analysis of data originally collected for the purpose of program management. The original data include information about CMC areas and non-CMC areas in all blocks that the CGPP works in (e.g., there was no sampling of sites within the CGPP program area). Given the type of data available for this paper, we chose a quasi-experimental uninterrupted time series analysis design with a nonequivalent comparison group [12]. This involves comparing CMC and non-CMC areas separately over time (each of the national immunization days during 2008-2009).

Program/intervention areas (CMC) and comparison areas (non-CMC) were not selected at random. CMC areas are a group of villages within a block that have been purposively selected because they have the most difficulty achieving vaccination program targets. CMC areas have a higher proportion of families that are resistant to allowing their children to be vaccinated with polio vaccine than non-CMC areas. Therefore, we would expect---in the absence of additional CGPP social mobilization efforts---that vaccination performance would be lower, on average, in CMC areas compared to non-CMC areas. However, if performance in CMC areas approaches or surpasses the performance in non-CMC areas, we might judge the social mobilization efforts as having had positive effects.

### Description of data

The original data represent programmatic monitoring data collected by CMCs during the course of their ongoing work as follows. In both CMC and non-CMC areas, government vaccinators from the Ministry of Health, supervised by their block level managers, tally standard information about the SIA (such as the number of children under five years who were vaccinated or the number of households with children under five remaining to be vaccinated). In CMC areas, the CMC is simultaneously collecting the same data. The CMCs maintain a detailed register that records the following: the number of children vaccinated at booths; the number of households visited after the booth day; and, the eventual immunization outcome at each house. Joint government and SM Net teams (BMCs and their block level government counterparts from the Ministry of Health) sit together and review vaccinators' tally sheets each night of the campaign. In addition, independent monitoring teams survey a percentage of houses after each SIA.

Each month, the CGPP CMC compiles her data and delivers a hard copy report to her supervisor, the BMC. The BMC compiles CMC data and submits it to the DMC who then sends a computerized report to the SRCs and the CGPP Secretariat. Data for non-CMC areas of each block were obtained by subtracting numbers for the CMC areas from the block's total (provided by the government). The CGPP Secretariat maintains a central database of these data. These data were then compiled and cleaned for this secondary data analysis.

The CGPP tracks three important indicators of social mobilization performance during SIAs: (1) Booth Coverage; (2) Percent of "X" households converted to "P" during both A and B team activities; and, (3) Percent total "X" households converted to "P" during B team activities (among those "X" households remaining after A team activities). These three indicators are considered sensitive to the social mobilization efforts of the CGPP CMCs and important milestones for vaccinating every child that needs it. Booth coverage is the proportion of eligible children that were vaccinated at vaccination booths. The denominator is the total number of children vaccinated during the previous SIA (at booths and during house-to-house visits by vaccinators), and therefore varies between SIAs. CMC activity should increase Booth Coverage by motivating families to take their children to vaccination booths located in their own communities. The definition of an "X" household is discussed above. CMC activity should also increase the Percent of "X" households converted to "P." The reasons for a household to have unvaccinated children (to be an "X" household) could include overt refusals by caregivers, sick children, children not present during vaccination, or locked houses. The CMC works hard to convince resistant parents and parents of sick children to get their children vaccinated.

The CGPP uses a database to maintain information about vaccination activities during SIAs. Each record in the database contains information about vaccination performance during a single SIA (organized by month) in one block. There is a separate record/row each representing the CMC areas in a block and the non-CMC areas in the same block. CMC areas cover roughly one third of the entire block while non-CMCs areas account for the remaining two-thirds. Record/rows in the database include the following information: district in which the SIA was conducted; block in which the SIA was conducted; whether the record represents a CMC or non-CMC area; the month and year of the SIA; the estimated number of children under age of five years living in the areas that the record represents; the three performance indicators described above; and, other data needed to calculate the three indicators. At the time of this writing, CGPP India was working in ten districts and the data has been consolidated for these districts from January 2008 through September 2009; data from earlier SIAs are in the database but are not considered to be of sufficient reliability or quality to include in the analysis.

### Exploratory Analysis

Exploratory analysis was carried out using Tableau Desktop visualization software [13]. Box plots and bar graphs were developed to compare the values of the three performance indicators described above between CMC and non-CMC areas. The values for each vaccination campaign by block and by CMC vs. non-CMC area were averaged for each district. The average indicator values for CMC vs. non-CMC areas were then compared by district.

### Statistical Analysis

We employed Generalized Estimating Equations (GEE) statistical method to account for the block level Intra-cluster Correlation (ICC), and used 'Quasi-likelihood under the independence model criterion (QIC)' as the model selection method. We considered the model with lowest QIC to be the most parsimonious model among the competing models with different correlation structures (exchangeable, auto-regressive, unstructured etc.).

We assumed that the differences between CMC and non-CMC areas for the indicators being assessed (booth coverage, conversion of X house to P during both A and B team activities, conversion of X houses to P during B team activities) might vary by district. We also assumed that an interaction between districts and these differences were possible. That is, we assumed the possibility that the differences between CMC and non-CMC areas may differ depending on the district being analyzed. To test these possibilities, we included the program district (and interaction terms if significant) along with CMC status in multi-variate statistical analysis using STATA statistical software, in addition to conducting bi-variate analysis (indicator vs. CMC status) [14].

## Results

### Exploratory Analysis

The districts and blocks included in this analysis (for the period 2008-2009) are listed in Table 1. Table 1 also show the number of vaccination campaigns each block contributes to the analysis. The number of campaigns per block ranges from seven to 17.

**Table 1:** Blocks included in the Analysis by District and Number of Polio Vaccination Campaigns, 2008-2009

**District**	**Block**	**Number of campaigns**	**District**	**Block**	**Number of campaigns**
	
Baghpat	Baghpat	15	Muzafarnagar	Baghra	17
	Baraut	15		Budhana	17
	Binauli	15		Charthawal	17
	Chaproli	15		Jansath	17
	Khekhra	15		Khatauli	17
	Pilana	15		Shamli	17
				Un	17
	
Bareilly	Baheri	17	Rampur	Bilaspur	17
	Bhojipura	17		Chamrua	17
	Dalelnagar	17		Swar	17
	Meerganj	17		Tanda	17
	Nawabganj	17			
	
Mau	Ghosi	15	Saharanpur	City	15
	Kopaganj	15		Nakur	15
	Pardaha	15		Sarsawan	15
	Ranipur	8		Sunehty	15
	Ratanpura	7			
	
Meerut	Hastinapur	17	Shahjahanpur	Bhawalkherha	17
	Kharkhauda	17		Jaitipur	17
	P. Garh	17		Kalan	17
	Rohta	17		Mirzapur	17
	Sardhana	17		Sindhauli	17
	
Moradabad	Bhojpur	17	Sitapur	Biswan	16
	M. Pandey	17		Machrehata	16
	Manota	17		Persendi	16
	Naroli	17		Pisawan	16
	Panwasa	17		Reusa	15
	Sambhal R	17		Sanda	15
	Sambhal U	17			
	Zone - 3	17			
	Zone - 4	17			
	Zone - 5	17			

**Figure 1:**
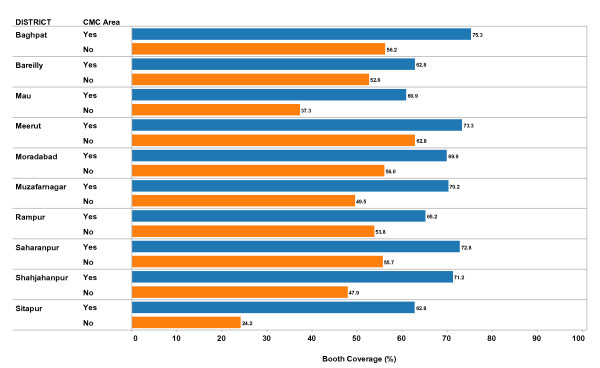
**Mean Booth Coverage (%) by District and CMC Status, 2008-2009**. Each bar represents the mean value for booth coverage during supplemental immunization activities (mass immunization campaigns) in the time period (2008-2009). The value is calculated at the block level separately for CMC areas and non-CMC areas. In this figure the mean values are calculated separately for each District, and separately for CMC areas compared to non-CMC areas. Blue bars represent CMC areas; Orange bars represent non-CMC areas. Booth coverage is the percent of all children living in a block (in either a CMC area or non-CMC area) who are vaccinated at a fixed vaccination post on the first day of the current vaccination campaign.

**Figure 2:**
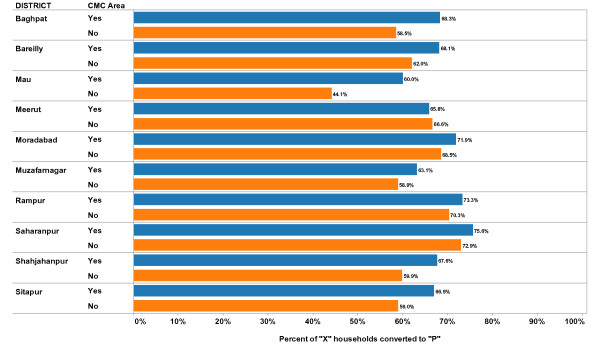
**Mean Percent of "X" Households Converted to "P" by District and CMC Status, 2008-2009**. Each bar represents the mean value for percent of "X" households converted to "P" during supplemental immunization activities (mass immunization campaigns) in the time period (2008-2009). The value is calculated at the block level separately for CMC areas and non-CMC areas. In this figure the mean values are calculated separately for each District, and separately for CMC areas compared to non-CMC areas. Blue bars represent CMC areas; Orange bars represent non-CMC areas. "X" households are those with children less than five years of age who were not vaccinated during the current vaccination campaign. A household is converted to "P" when all children less than five years of age in the household is vaccinated during the current vaccination campaign.

**Figure 3:**
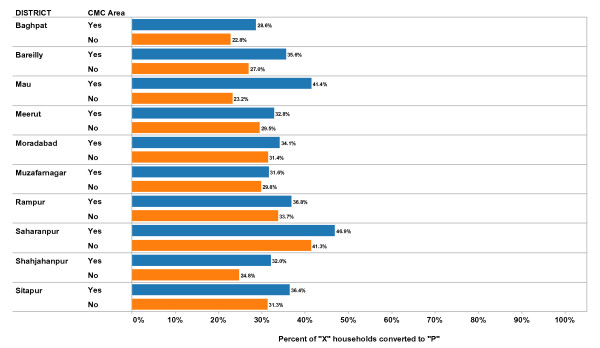
**Mean Percent of "X" Households Converted to "P" during B Team Phase by District and CMC Status, 2008-2009**. Each bar represents the mean value for percent of "X" households converted to "P" during supplemental immunization activities (mass immunization campaigns) in the time period (2008-2009) during what is called the B Team phase. The value is calculated at the block level separately for CMC areas and non-CMC areas. In this figure the mean values are calculated separately for each District, and separately for CMC areas compared to non-CMC areas. Blue bars represent CMC areas; Orange bars represent non-CMC areas. "X" households are those with children less than five years of age who were not vaccinated during the current vaccination campaign. A household is converted to "P" when all children less than five years of age in the household is vaccinated during the current vaccination campaign. The B Team phase represents a second attempt to vaccinate children by going house to house. Prior to the B Team phase, A teams have gone house to house to vaccinate children and have marked houses with an "X" if a child less than five years of age lives in the house but remains unvaccinated.

Figures 1, 2 and 3 include the main results of our exploratory analysis. In Figure 1, we compare mean booth coverage between CMC and non-CMC areas for each district. That is, the booth coverage in each block for each vaccination campaign was average separately for CMC areas and non-CMC areas by district for all campaigns carried out in 2008 and 2009. In each district, the mean booth coverage in CMC areas is higher than in non-CMC areas. The difference in booth coverage between CMC and non-CMC areas varies greatly by district suggesting a possible interaction: the effect on booth coverage due to CMC status is modified by which district we analyze. In Sitapur District, the average difference in booth coverage between CMC and non-CMC areas is almost 40%; in Bareilly the difference is 10%.

Figure 2 shows the mean percent of "X" households that were converted to "P" during the vaccination campaign by district and by CMC vs. non-CMC areas. The mean values represent the average values of each block in the district for all campaigns carried out in 2008 and 2009. In all districts except one (Meerut), the percent of X households converted to P was higher in CMC areas, on average, than in non-CMC areas. The differences between CMC and non-CMC areas varied by district from 15% (Baghpat) to almost -1% (Meerut).

The mean percent of "X" households that were converted to "P" during the B team activities is shown in Figure 3 by district and by CMC vs. non-CMC areas. The mean values represent the average values of each block in the district for all campaigns carried out in 2008 and 2009. In all districts, the percent of X households converted to P during the B team activities was higher in CMC areas, on average, than in non-CMC areas. The differences between CMC and non-CMC areas varied by district from 18% (Mau) to 2% (Muzafarnagar).

### Statistical Analysis

#### Booth Coverage

The values of a bi-variate analysis of mean booth coverage vs. CMC status, adjusting for clustering by block, is shown at the top of Table 2. On average, booth coverage in CMC areas is 69% vs. 50% for non-CMC areas---a difference of about 19% higher in CMC areas. The difference is statistically significant.

**Table 2:** Mean Booth Coverage by District and CMC Status, 2008-2009

	Booth Coverage (%) (95% Confidence Interval)		
	Non-CMC Areas (n = 56)	CMC Areas (n = 56)	P-Value
All Districts	49.8 (46.5, 53.1)	68.8 (66.9, 70.6)	< .001*, **
By District			
Baghpat	56.2 (53.0, 59.4)	75.3 (72.3, 78.2)	< .001**
Bareilly	52.6 (48.7, 56.5)	62.8 (56.5, 69.1)	< .05**
Mau	37.2 (34.7, 39.9)	60.9 (57.5, 64.2)	< .001**
Meerut	62.8 (56.4, 69.3)	73.3 (66.9, 79.8)	< .05**
Moradabad	56.0 (49.7, 62.3)	69.9 (65.8, 74.0)	< .001**
Muzafarnagar	49.5 (46.5, 52.5)	70.2 (66.6, 73.9)	< .001**
Rampur	53.8 (52.4, 55.2)	65.2 (62.5, 67.9)	< .001**
Saharanpur	55.7 (49.8, 61.5)	72.8 (66.5, 79.1)	< .001**
Shahjahanpur	47.9 (41.3, 54.5)	71.2 (68.9, 73.4)	< .001**
Sitapur	24.2 (21.4, 27.0)	62.8 (59.8, 65.7)	< .001**

* Bivariate analysis of Booth Coverage by CMC Status using generalized estimating equations (standard errors adjusted for clustering by block/CMC Status)

** Multivariate Analysis of Booth Coverage by CMC Status controlling for District, and including interaction of District with CMC Status, using generalized estimating equations (standard errors adjusted for clustering by block/CMC Status)

Table 2 also includes the results of a multi-variate analysis that controls for the district as well as the interaction between CMC status and district; the interaction was statistically significant. For this reason, we provide the district level values for mean booth coverage for CMC areas vs. Non-CMC areas. The amount of the difference between CMC and non-CMC areas varies across districts. In all districts, the CMC areas had significantly higher mean booth coverage than in non-CMC areas: the difference ranged from about 10% to almost 40%. The overall mean booth coverage (all districts combined) was statistically significantly higher in CMC areas compared to non-CMC areas in the multi-variate analysis, as was found in the bi-variate analysis.

#### X to P Conversion

The top of Table 3 provides the values of the bi-variate analysis of the mean percent of 'X' houses converted to 'P' by CMC Status. The difference between CMC areas and non-CMC areas is statistically significant. On average, the mean percent in CMC areas is 68% vs. 62% for non-CMC areas.

**Table 3:** Percent of 'X' Houses Converted to 'P' by District and CMC Status, 2008-2009

	Conversion of X to P (%) (95% Confidence Interval)		
	Non-CMC Areas (n = 56)	CMC Areas (n = 56)	P-Value
All Districts	62.3 (59.9, 64.7)	67.9 (66.0, 69.8)	< .001*,**
By District			
Baghpat	58.2 (54.8, 61.5)	68.0 (64.5, 71.7)	< .001**
Bareilly	61.6 (60.4, 62.9)	68.0 (61.6, 74.3)	
Mau	44.1 (39.8, 48.5)	60.0 (53.7, 66.3)	< .001**
Meerut	66.6 (62.8, 70.5)	65.7 (61.8, 69.6)	
Moradabad	68.2 (62.0, 74.5)	71.7 (65.9, 77.5)	
Muzafarnagar	58.2 (52.9, 63.6)	62.7 (57.8, 67.6)	
Rampur	70.1 (66.8, 73.4)	73.1 (69.3, 77.0)	
Saharanpur	72.9 (69.8, 76.1)	75.6 (73.3, 77.9)	
Shahjahanpur	59.9 (53.0, 66.7)	67.5 (63.5, 71.5)	
Sitapur	59.0 (57.1, 60.8)	66.9 (65.2, 68.7)	< .001**

* Bivariate analysis of X-to-P Conversion by CMC Status using generalized estimating equations (standard errors adjusted for clustering by block/CMC Status)

** Multivariate Analysis of X-to-P Conversion by CMC Status controlling for District, and including interaction of District with CMC Status, using generalized estimating equations (standard errors adjusted for clustering by block/CMC Status)

Table 3 also includes the results of a multi-variate analysis that controls for district as well as the interaction between CMC status and district. Table 3 shows the district level values for percent of 'X' houses converted to 'P'. The amount of the difference between CMC and non-CMC areas varies across districts. In three districts, the CMC areas had a significantly higher mean percent than in non-CMC areas. In six districts, the mean percent was higher in CMC areas than in non-CMC areas, but the difference was not statistically significant to the 0.05 level. In one district (Meerut as suggested by the exploratory data analysis) the mean percent was higher in non-CMC areas (67%) than in CMC areas (66%). The overall mean percent of 'X' houses converted to 'P' (all districts combined and controlling for district and interaction effects) was statistically significantly higher in CMC areas compared to non-CMC areas in the multi-variate analysis, as was found in the bi-variate analysis.

#### X to P Conversion during B Team activities

The values of the bi-variate analysis of the mean percent of 'X' houses converted to 'P' during the B team activities by CMC status are shown in Table 4. The difference between CMC areas and non-CMC areas is statistically significant. On average, the mean percent in CMC areas is 35% vs. 29% for non-CMC areas.

**Table 4:** Percent of 'X' Houses Converted to 'P' during "B-Phase" by District and CMC Status, 2008-2009

	Conversion of X to P B-Phase (%) (95% Confidence Interval)		
	Non-CMC Areas (n = 56)	CMC Areas (n = 56)	P-Value
All Districts	29.3 (27.2, 31.5)	34.7 (32.7, 36.8)	< .001*,**
By District			
Baghpat	23.2 (20.6, 25.8)	28.7 (25.9, 31.5)	< .05**
Bareilly	28.3 (24.4, 32.1)	33.7 (29.2, 38.2)	< .05**
Mau	29.6 (22.3, 36.8)	35.0 (27.4, 42.7)	< .01**
Meerut	28.4 (25.0, 31.8)	33.9 (30.5, 37.3)	
Moradabad	29.9 (25.7, 34.2)	35.4 (31.7, 39.1)	
Muzafarnagar	27.4 (23.1, 31.8)	32.9 (28.4, 37.3)	
Rampur	32.5 (29.6, 35.5)	38.0 (34.9, 41.1)	
Saharanpur	41.4 (37.0, 45.7)	46.8 (42.6, 51.1)	
Shahjahanpur	25.7 (20.7, 30.7)	31.1 (26.7, 35.6)	
Sitapur	31.1 (27.9, 34.3)	36.6 (33.3, 39.9)	

* Bivariate analysis of X-to-P Conversion by CMC Status using generalized estimating equations (standard errors adjusted for clustering by block/CMS Status)

** Multivariate Analysis of X-to-P Conversion by CMC Status controlling for District, using generalized estimating equations (standard errors adjusted for clustering by block/CMS Status)

Table 4 also includes the results of a multi-variate analysis that controls for district effects. [NB: the interaction effect between CMC status and district was not significant and not included in this analysis]. In three districts, the CMC areas had a significantly higher mean percent than in non-CMC areas. In seven districts, the mean percent was higher in CMC areas than in non-CMC areas, but the difference was not statistically significant to the 0.05 level. The overall mean percent of 'X' houses converted to 'P' during B team activities (all districts combined and controlling for district effects) was statistically significantly higher in CMC areas compared to non-CMC areas in the multi-variate analysis, as was found in the bi-variate analysis.

## Discussion

### Limitations

The data and analysis have several limitations. First, we were unable to carry out an experimental design. Program/intervention areas (CMC) and comparison areas (non-CMC) were not selected at random. CMC areas are a group of villages within a block that have been purposively selected because they have the most difficulty achieving vaccination program targets. They tend to have a much higher proportion of families that are resistant to allowing their children to be vaccinated with polio vaccine. Therefore, it would be understandable if values of the three performance indicators selected would be lower, on average, in CMC areas compared to non-CMC areas even after considerable social mobilization effort. However, if performance in CMC areas approaches or surpasses the performance in non-CMC areas, we might judge the social mobilization efforts as having had positive effects.

Another limitation of the data is the difficulty of analyzing trends. We expect performance to vary up and down by season. For example, during harvest seasons we might find fewer household members and eligible children at home; the number of vaccinations would decrease in this situation while the denominator would remain high. In addition, the denominator (number of eligible children under five years of age to be vaccinated) changes from SIA to SIA. The denominator is the number of children under five vaccinated during the prior campaign. We assume this number to be a more accurate estimate of the actual number of children under five living in the area than the census because health workers going house-to-house on a regular basis to count this number of children obtain this number. In this situation, we would expect vaccination performance to move up and down from SIA to SIA as an artifact of the changing denominator. For this reason, the analysis looked at the mean difference in performance between CMC and non-CMC areas rather than comparing trends between the two comparison groups.

There is limitation in assessing the effects of social mobilization on performance of supplementary immunization activities such as national or sub-national immunization days. SIAs, while necessary, are not sufficient. Many other factors affect progress of the polio eradication effort such as routine immunization efforts, sanitation, and vaccine efficacy in crowded, unsanitary areas.

### Booth Coverage

Booth coverage in CMC areas is clearly higher than in non-CMC areas, even though we expect it to be more difficult to vaccinate in villages in CMC areas as compared to non-CMC areas. This finding was consistent across all districts in the analysis. A very likely explanation, considering, is that the social mobilization efforts of the SM Net in the CGPP areas are responsible for much of the increased booth coverage in CMC areas as compared to non-CMC efforts.

### Conversion of "X" Households to "P"

Even though we expect it to be more difficult to vaccinate in villages in CMC areas as compared to non-CMC areas, performance on these two indicators appears to validate the social mobilization efforts of the SM Net in the CGPP Project. In all but one instance in one district, the percent of "X" households converted to "P" was higher in CMC areas than in non-CMC areas. In several districts, the percent was even significantly higher in CMC areas vs. non-CMC areas. [Note that in the one instance where the percent was lower in CMC areas, the percent was not significantly lower statistically]. This suggests that the predicted result of SM Net social mobilization activities is an increased number of families allowing their children to be vaccinated after first refusing, even in villages that have a high number of families resistant to polio vaccination; that these difficulties can be overcome.

### SM Net and CORE Group Polio Project (CGPP)

The SM Net approach meets the Global Polio Eradication Initiative recommendation to reach underserved populations and improve communications and social mobilization in priority areas (personal communication). In this paper, we only reviewed the vaccination outcomes in the CGPP India program areas of the SM Net. The CGPP approach is to achieve scale of effort in a coordinated way, through a partnership of PVOs and NGOs under the leadership of an independent Secretariat. While it may be assumed that PVO and NGO programs are limited in scale to a few communities, the CGPP India covered 10 districts and over 1000 geographic areas. The vaccination outcomes in CGPP program areas met or exceeded the vaccination outcomes in non-program areas---even though program areas were purposively selected because the challenges vaccinating in these areas were greater. This is suggestive of the added-value of CGPP social mobilization efforts. We can develop an evidence-based hypothesis that vaccination outcomes will be higher/better in CGPP program areas, other things being equal. We might assume that we would find similar performance in other SM Net program areas, but this should be confirmed.

### Policy Implications

In populations that are resistant to public health programs, additional social mobilization efforts should be carried out. The SM Net and CGPP provide a model for social mobilization that should be considered as new social mobilization approaches are designed. This includes developing a partnership between the government, non-governmental organizations, available and interested multi-lateral organizations and donors. A partnership with NGOs via coordinating/leadership body like the CGPP allows for significant scale up of NGO activities. Further research should be supported to identify the specific social mobilization activities that provide the greatest benefit.

## Conclusions

The SM Net was established in the northern Indian state of Uttar Pradesh---in high-risk villages and urban areas in high transmission areas of a country endemic for polio---for the purpose of motivating families to have all their children under five years of age receive polio vaccine during routine immunization services and during supplemental immunization activities (SIAs). Even though we expect it to be more difficult to vaccinate in SM Net villages as compared to non-SM Net villages, performance was as good and often better in CGPP programs areas vs. non-CGPP program areas. This was observed even though villages in CGPP program areas were purposively selected because they had a high proportion of families (relative to villages in non-CGPP program areas) that were resistant to having their children vaccinated against polio. The performance of the CGPP Project on indicators assessed appears to validate the social mobilization efforts of the SM Net in India toward eradication of polio. The CGPP model---achieving scale through a partnership of PVOs and NGOs under the leadership of an independent Secretariat---appears promising and should be explored further as a model for social mobilization and other health programming efforts. Additional analysis as to what particular social mobilization efforts are associated with better performance on the vaccination indicators included in this analysis is recommended.

## Competing interests

All authors have received salary support from the US Agency for International Development (USAID) under Cooperative Agreement GHN-A-00-07-00014. This salary support has covered implementation of the project described and/or for writing this manuscript.

## Authors' contributions

All authors have read and approved the final version of the manuscript. WW wrote key sections of the Methods, Results, Discussions and Conclusions. He also designed and carried out exploratory and statistical analysis. HR wrote key sections of the Methods and assisted with analysis of longitudinal data. RS wrote key sections of the Background and edited the manuscript. VS compiled the data for analysis, helped write the Background, and edited the manuscript. DW edited the manuscript and assisted in the design of the analysis.

## Pre-publication history

The pre-publication history for this paper can be accessed here:

http://www.biomedcentral.com/1471-2334/11/117/prepub
